# Supplementary material for: Characterisation of Cytotoxicity-Related Receptors on γδ T Cells in Chronic Lymphocytic Leukaemia
Source: Cells. 2025 Mar 18;14(6):451. doi: 10.3390/cells14060451 (PMC11941621; doi:10.3390/cells14060451)
Supplement: Supplementary file 1 [file cells-14-00451-s001.zip › cells-3467349-supplementary.pdf]

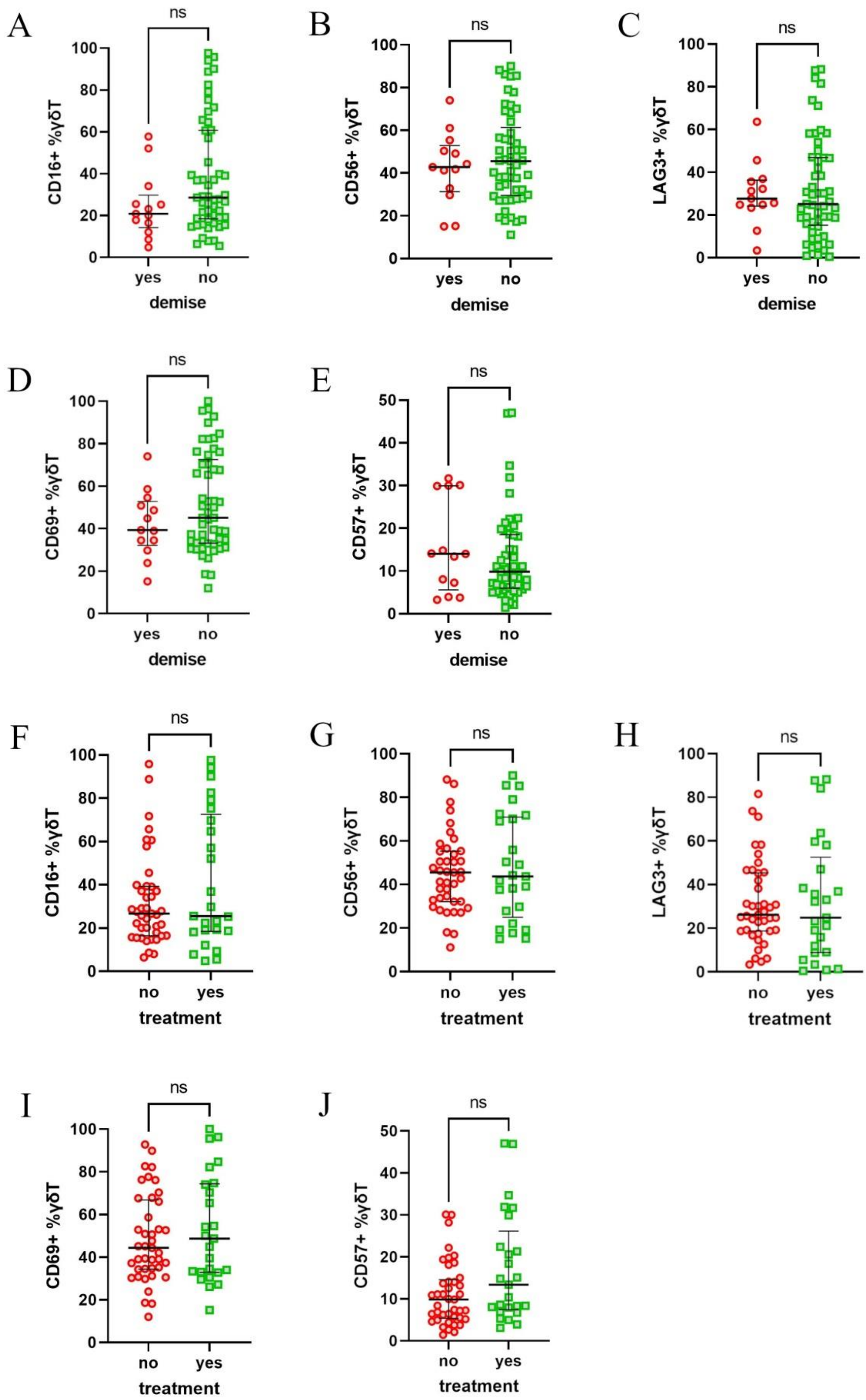

Supp. Figure S1. Comparison of CD16, CD56, CD57, CD69 and LAG-3 expression on γδ T cells in CLL patients subdivided based on the treatment requirement and whether they survived the observational period.

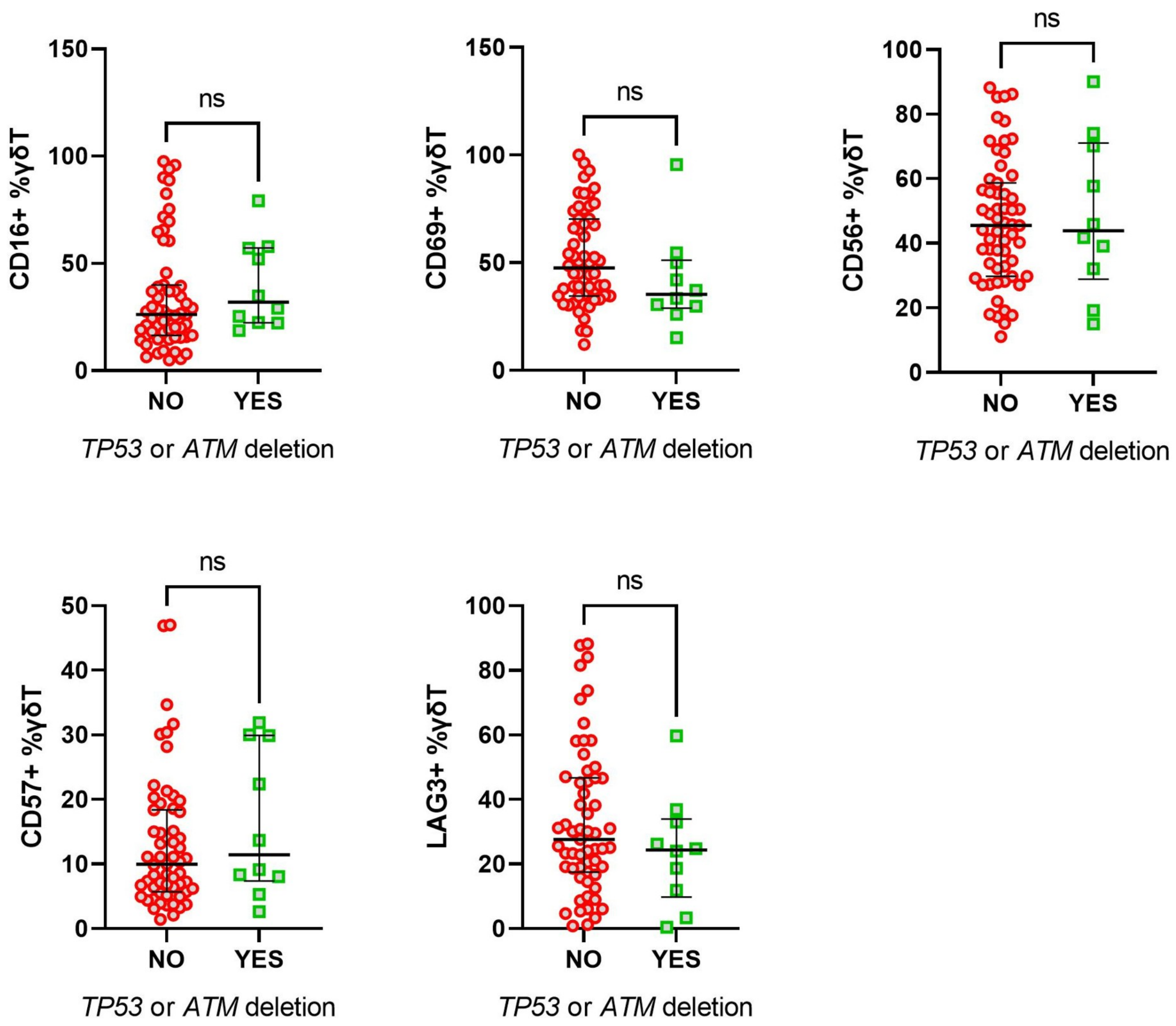

Supp. Figure S2. Comparison of γδ T cell marker expression between CLL patients with and without TP53 or ATM deletion. Scatter plots display the percentage of γδ T cells expressing CD16, CD69, CD56, CD57, and LAG3, with no significant differences (ns) observed between the groups.

Supp. Table S1. Sociodemographical and clinical characteristics of CLL patients and healthy controls

	CLL patients	Healthy Volunteers (HV)
Sex (χ^2 test: p = 0.966)	F: 43.48% (N=30)<br>M: 56.52% (N=39)	F: 42.857% (N=6)<br>M: 57.143% (N=8)
Age [Mean ± SD] (Mann-Whitney U test: p = 0.001)	66.71±8.94	58.917±9.42
Treatment needed	No: 62.3% (N=43)<br>Yes: 37.7% (N=26)	
Demise during observation	No: 18.8% (N=13)<br>Yes: 81.2% (N=56)	
Rai stadium	0: 44.1% (N=30)<br>1: 17.6% (N=12)<br>2: 25.0% (N=17)<br>3: 4.4% (N=3)<br>4: 8.8% (N=6)	
ZAP-70 expression (Cut-off: 20%)	low: 84.6% (N=55)<br>high: 15.4% (N=10)	
CD38 expression (Cut-off: 30%)	low: 72.3% (N=47)<br>high: 27.7% (N=18)	
Cytogenetical ablities	No: 82.4% (N=42)<br>Yes: 17.6% (N=9)	

Supp. Table S2. The detailed configuration of cytometer

Beckman Coulter Cytoflex LX			
No.	Laser	Filter setup	Detector name
1.	375 nm	450/45	NUV450
2.		525/40	NUV525
3.		675/30	NUV675
4.	405 nm	450/45	Pacific Blue
5.		525/40	Krome Orange
6.		610/20	V610
7.		660/10	V660
8.		763/43	V763
9.	488 nm	525/40	FITC
10.		610/20	ECD
11.		690/50	PE-Cy5.5
12.	561 nm	585/42	PE
13.		610/20	mCherry
14.		675/30	PE-Cy5
15.		710/50	PE-Cy5.5
16.		763/43	PE-Cy7
17.	638 nm	660/10	APC
18.		712/25	APC-A700
19.		763/43	APC-A750
20.	808 nm	840/20	Alexa Fluor 790
21.		885/40	IR885

Supp. Table S3. Full correlation matrix

Spearman's Correlations

			Spearman's rho	p
CD57	-	CD69	0.073	0.512
CD57	-	LAG	0.058	0.605
CD57	-	CD16	0.314**	0.004
CD57	-	CD56	0.305**	0.005
CD57	-	AGE	0.160	0.188
CD57	-	TTT (months)	-0.297	0.217
CD57	-	WBC	-0.162	0.194
CD57	-	LYMPH	-0.077	0.539
CD57	-	Monocytes [K/ul]	-0.034	0.786
CD57	-	Mon %	0.048	0.702
CD57	-	Neutr [K/ul] [2,8- 6,8K/ul]	-0.290*	0.019
CD57	-	Neutr % [48,7 - 70,1%]	-0.027	0.833
CD57	-	HGB	-0.236	0.057
CD57	-	PLT	-0.249*	0.046
CD57	-	B2MICR	0.072	0.578
CD57	-	LDH	-0.038	0.766
CD57	-	IGA	-0.280*	0.049
CD57	-	IGG	-0.261	0.065
CD57	-	IGM	-0.214	0.135
CD57	-	CD19%	0.004	0.974
CD57	-	CD5_19%	0.123	0.331
CD69	-	LAG	0.205	0.065
CD69	-	CD16	0.063	0.570
CD69	-	CD56	-0.006	0.954
CD69	-	AGE	0.359**	0.002
CD69	-	TTT (months)	0.174	0.477
CD69	-	WBC	0.106	0.396
CD69	-	LYMPH	0.159	0.203
CD69	-	Monocytes [K/ul]	0.069	0.584
CD69	-	Mon %	-0.023	0.855
CD69	-	Neutr [K/ul] [2,8- 6,8K/ul]	0.043	0.731
CD69	-	Neutr % [48,7 - 70,1%]	-0.152	0.226
CD69	-	HGB	-0.144	0.247
CD69	-	PLT	-0.049	0.697
CD69	-	B2MICR	-0.073	0.575
CD69	-	LDH	0.101	0.429
CD69	-	IGA	0.030	0.836
CD69	-	IGG	-0.075	0.601
CD69	-	IGM	0.085	0.559
CD69	-	CD19%	0.115	0.363
CD69	-	CD5_19%	-0.001	0.991
LAG	-	CD16	-0.272*	0.013
LAG	-	CD56	0.162	0.146
LAG	-	AGE	-0.030	0.805
LAG	-	TTT (months)	-0.175	0.472
LAG	-	WBC	0.141	0.258
LAG	-	LYMPH	0.223	0.071
LAG	-	Monocytes [K/ul]	-0.060	0.637
LAG	-	Mon %	-0.297*	0.016
LAG	-	Neutr [K/ul] [2,8- 6,8K/ul]	0.025	0.844
LAG	-	Neutr % [48,7 - 70,1%]	-0.232	0.063
LAG	-	HGB	-0.105	0.400
LAG	-	PLT	-0.016	0.899

Spearman's Correlations

			Spearman's rho	p
LAG	-	B2MICR	-0.078	0.546
LAG	-	LDH	0.161	0.204
LAG	-	IGA	-0.166	0.251
LAG	-	IGG	-0.190	0.181
LAG	-	IGM	-0.215	0.134
LAG	-	CD19%	0.228	0.067
LAG	-	CD5_19%	0.286*	0.021
CD16	-	CD56	0.316**	0.004
CD16	-	AGE	0.206	0.089
CD16	-	TTT (months)	0.209	0.391
CD16	-	WBC	-0.134	0.285
CD16	-	LYMPH	-0.062	0.621
CD16	-	Monocytes [K/ul]	-0.111	0.378
CD16	-	Mon %	-0.029	0.817
CD16	-	Neutr [K/ul] [2,8- 6,8K/ul]	-0.338**	0.006
CD16	-	Neutr % [48,7 - 70,1%]	-0.077	0.544
CD16	-	HGB	0.026	0.834
CD16	-	PLT	-0.276*	0.026
CD16	-	B2MICR	0.187	0.145
CD16	-	LDH	-0.597***	< .001
CD16	-	IGA	0.049	0.736
CD16	-	IGG	0.075	0.599
CD16	-	IGM	0.201	0.161
CD16	-	CD19%	-0.122	0.333
CD16	-	CD5_19%	-0.240	0.054
CD56	-	AGE	-0.003	0.979
CD56	-	TTT (months)	-0.176	0.470
CD56	-	WBC	-0.214	0.085
CD56	-	LYMPH	-0.203	0.102
CD56	-	Monocytes [K/ul]	-0.032	0.798
CD56	-	Mon %	0.102	0.417
CD56	-	Neutr [K/ul] [2,8- 6,8K/ul]	-0.085	0.502
CD56	-	Neutr % [48,7 - 70,1%]	0.124	0.326
CD56	-	HGB	0.055	0.658
CD56	-	PLT	-0.096	0.449
CD56	-	B2MICR	-0.059	0.648
CD56	-	LDH	-0.141	0.265
CD56	-	IGA	-0.045	0.756
CD56	-	IGG	-0.074	0.607
CD56	-	IGM	-0.012	0.933
CD56	-	CD19%	0.045	0.724
CD56	-	CD5_19%	-0.088	0.484
AGE	-	TTT (months)	0.227	0.350
AGE	-	WBC	0.011	0.928
AGE	-	LYMPH	-0.029	0.818
AGE	-	Monocytes [K/ul]	0.184	0.143
AGE	-	Mon %	0.310*	0.012
AGE	-	Neutr [K/ul] [2,8- 6,8K/ul]	-0.253*	0.042
AGE	-	Neutr % [48,7 - 70,1%]	-0.084	0.504
AGE	-	HGB	-0.406***	< .001
AGE	-	PLT	-0.401***	< .001
AGE	-	B2MICR	0.272*	0.033
AGE	-	LDH	0.006	0.963
AGE	-	IGA	-0.053	0.714
AGE	-	IGG	0.137	0.336
AGE	-	IGM	0.131	0.364

Spearman's Correlations

			Spearman's rho	p
AGE	-	CD19%	0.047	0.708
AGE	-	CD5_19%	-0.017	0.893
TTT (months)	-	WBC	0.168	0.505
TTT (months)	-	LYMPH	0.192	0.445
TTT (months)	-	Monocytes [K/ul]	0.432	0.073
TTT (months)	-	Mon %	0.102	0.688
TTT (months)	-	Neutr [K/ul] [2,8- 6,8K/ul]	-0.070	0.784
TTT (months)	-	Neutr % [48,7 - 70,1%]	-0.361	0.141
TTT (months)	-	HGB	0.096	0.706
TTT (months)	-	PLT	-0.166	0.511
TTT (months)	-	B2MICR	0.093	0.731
TTT (months)	-	LDH	0.060	0.818
TTT (months)	-	IGA	-0.329	0.353
TTT (months)	-	IGG	-0.590	0.056
TTT (months)	-	IGM	-0.352	0.319
TTT (months)	-	CD19%	0.107	0.682
TTT (months)	-	CD5_19%	-0.123	0.637
WBC	-	LYMPH	0.916***	< .001
WBC	-	Monocytes [K/ul]	0.518***	< .001
WBC	-	Mon %	-0.309*	0.012
WBC	-	Neutr [K/ul] [2,8- 6,8K/ul]	0.250*	0.044
WBC	-	Neutr % [48,7 - 70,1%]	-0.789***	< .001
WBC	-	HGB	-0.306*	0.012
WBC	-	PLT	-0.193	0.124
WBC	-	B2MICR	0.370**	0.003
WBC	-	LDH	0.217	0.085
WBC	-	IGA	-0.240	0.093
WBC	-	IGG	-0.180	0.206
WBC	-	IGM	-0.356*	0.011
WBC	-	CD19%	0.649***	< .001
WBC	-	CD5_19%	0.355**	0.004
LYMPH	-	Monocytes [K/ul]	0.477***	< .001
LYMPH	-	Mon %	-0.400***	< .001
LYMPH	-	Neutr [K/ul] [2,8- 6,8K/ul]	0.169	0.178
LYMPH	-	Neutr % [48,7 - 70,1%]	-0.895***	< .001
LYMPH	-	HGB	-0.244*	0.048
LYMPH	-	PLT	-0.273*	0.028
LYMPH	-	B2MICR	0.255*	0.045
LYMPH	-	LDH	0.213	0.091
LYMPH	-	IGA	-0.350*	0.013
LYMPH	-	IGG	-0.199	0.162
LYMPH	-	IGM	-0.339*	0.016
LYMPH	-	CD19%	0.666***	< .001
LYMPH	-	CD5_19%	0.436***	< .001
Monocytes [K/ul]	-	Mon %	0.531***	< .001
Monocytes [K/ul]	-	Neutr [K/ul] [2,8- 6,8K/ul]	0.191	0.128
Monocytes [K/ul]	-	Neutr % [48,7 - 70,1%]	-0.441***	< .001
Monocytes [K/ul]	-	HGB	-0.341**	0.005
Monocytes [K/ul]	-	PLT	-0.335**	0.007
Monocytes [K/ul]	-	B2MICR	0.318*	0.013
Monocytes [K/ul]	-	LDH	0.345**	0.006
Monocytes [K/ul]	-	IGA	-0.344*	0.016
Monocytes [K/ul]	-	IGG	-0.275	0.053
Monocytes [K/ul]	-	IGM	-0.351*	0.013
Monocytes [K/ul]	-	CD19%	0.369**	0.003
Monocytes [K/ul]	-	CD5_19%	0.111	0.391

Spearman's Correlations

			Spearman's rho	p
Mon %	-	Neutr [K/ul] [2,8- 6,8K/ul]	-0.063	0.620
Mon %	-	Neutr % [48,7 - 70,1%]	0.321**	0.009
Mon %	-	HGB	-0.181	0.150
Mon %	-	PLT	-0.209	0.097
Mon %	-	B2MICR	0.112	0.392
Mon %	-	LDH	0.119	0.351
Mon %	-	IGA	-0.004	0.980
Mon %	-	IGG	-0.059	0.683
Mon %	-	IGM	0.087	0.553
Mon %	-	CD19%	-0.285*	0.025
Mon %	-	CD5_19%	-0.277*	0.030
Neutr [K/ul] [2,8- 6,8K/ul]	-	Neutr % [48,7 - 70,1%]	0.226	0.070
Neutr [K/ul] [2,8- 6,8K/ul]	-	HGB	0.241	0.053
Neutr [K/ul] [2,8- 6,8K/ul]	-	PLT	0.474***	< .001
Neutr [K/ul] [2,8- 6,8K/ul]	-	B2MICR	-0.073	0.577
Neutr [K/ul] [2,8- 6,8K/ul]	-	LDH	0.348**	0.005
Neutr [K/ul] [2,8- 6,8K/ul]	-	IGA	0.174	0.232
Neutr [K/ul] [2,8- 6,8K/ul]	-	IGG	-0.065	0.656
Neutr [K/ul] [2,8- 6,8K/ul]	-	IGM	-0.077	0.598
Neutr [K/ul] [2,8- 6,8K/ul]	-	CD19%	0.248	0.052
Neutr [K/ul] [2,8- 6,8K/ul]	-	CD5_19%	0.172	0.181
Neutr % [48,7 - 70,1%]	-	HGB	0.400***	< .001
Neutr % [48,7 - 70,1%]	-	PLT	0.519***	< .001
Neutr % [48,7 - 70,1%]	-	B2MICR	-0.318*	0.013
Neutr % [48,7 - 70,1%]	-	LDH	-0.084	0.511
Neutr % [48,7 - 70,1%]	-	IGA	0.407**	0.004
Neutr % [48,7 - 70,1%]	-	IGG	0.102	0.481
Neutr % [48,7 - 70,1%]	-	IGM	0.285*	0.047
Neutr % [48,7 - 70,1%]	-	CD19%	-0.545***	< .001
Neutr % [48,7 - 70,1%]	-	CD5_19%	-0.343**	0.006
HGB	-	PLT	0.543***	< .001
HGB	-	B2MICR	-0.595***	< .001
HGB	-	LDH	-0.177	0.161
HGB	-	IGA	0.335*	0.017
HGB	-	IGG	0.127	0.373
HGB	-	IGM	0.259	0.070
HGB	-	CD19%	-0.210	0.099
HGB	-	CD5_19%	-0.061	0.634
PLT	-	B2MICR	-0.423***	< .001
PLT	-	LDH	-0.015	0.904
PLT	-	IGA	0.446**	0.001
PLT	-	IGG	0.163	0.257
PLT	-	IGM	0.181	0.213
PLT	-	CD19%	-0.113	0.382
PLT	-	CD5_19%	-0.178	0.166
B2MICR	-	LDH	0.031	0.815
B2MICR	-	IGA	-0.258	0.074
B2MICR	-	IGG	-0.024	0.869
B2MICR	-	IGM	-0.184	0.205
B2MICR	-	CD19%	0.156	0.239
B2MICR	-	CD5_19%	0.029	0.826
LDH	-	IGA	-0.146	0.322
LDH	-	IGG	-0.170	0.243
LDH	-	IGM	-0.156	0.289
LDH	-	CD19%	0.240	0.062
LDH	-	CD5_19%	0.197	0.128

Spearman's Correlations

			Spearman's rho	p
IGA	-	IGG	0.511***	< .001
IGA	-	IGM	0.510***	< .001
IGA	-	CD19%	-0.140	0.338
IGA	-	CD5_19%	-0.026	0.860
IGG	-	IGM	0.524***	< .001
IGG	-	CD19%	0.018	0.902
IGG	-	CD5_19%	-0.041	0.780
IGM	-	CD19%	-0.182	0.210
IGM	-	CD5_19%	-0.063	0.667
CD19%	-	CD5_19%	0.578***	< .001

* p < .05, ** p < .01, *** p < .001
